# Postoperative [^68^Ga]Ga-DOTA-TATE PET/CT imaging is prognostic for progression-free survival in meningioma WHO grade 1

**DOI:** 10.1007/s00259-023-06400-3

**Published:** 2023-08-29

**Authors:** Nico Teske, Annamaria Biczok, Stefanie Quach, Franziska J. Dekorsy, Robert Forbrig, Raphael Bodensohn, Maximilian Niyazi, Joerg-Christian Tonn, Nathalie L. Albert, Christian Schichor, Moritz Ueberschaer

**Affiliations:** 1 Department of Neurosurgery, LMU University Hospital, LMU Munich, Marchioninistrasse 15, 81377 Munich, Germany; 2 German Cancer Consortium (DKTK), Partner Site Munich, Munich, Germany; 3 Department of Nuclear Medicine, LMU University Hospital, LMU Munich, Munich, Germany; 4 Institute of Neuroradiology, LMU University Hospital, LMU Munich, Munich, Germany; 5 Department of Radiation Oncology, LMU University Hospital, LMU Munich, Munich, Germany; 6 Bavarian Center for Cancer Research (BZKF), Erlangen, Germany

**Keywords:** Meningioma, Somatostatin receptor, PET, Extent of resection, Recurrence, Radiotherapy planning

## Abstract

**Purpose:**

Tumor resection represents the first-line treatment for symptomatic meningiomas, and the extent of resection has been shown to be of prognostic importance. Assessment of tumor remnants with somatostatin receptor PET proves to be superior to intraoperative estimation with Simpson grading or MRI. In this preliminary study, we evaluate the prognostic relevance of postoperative PET for progression-free survival in meningiomas.

**Methods:**

We conducted a post hoc analysis on a prospective patient cohort with resected meningioma WHO grade 1. Patients received postoperative MRI and [^68^Ga]Ga-DOTA-TATE PET/CT and were followed regularly with MRI surveillance scans for detection of tumor recurrence/progression.

**Results:**

We included 46 patients with 49 tumors. The mean age at diagnosis was 57.8 ± 1.7 years with a male-to-female ratio of 1:1.7. Local tumor progression occurred in 7/49 patients (14%) after a median follow-up of 52 months. Positive PET was associated with an increased risk for progression (*p = 0.015) and a lower progression-free survival (*p = 0.029), whereas MRI was not. 20 out of 20 patients (100%) with negative PET findings remained recurrence-free. The location of recurrence/progression on MRI was adjacent to regions where postoperative PET indicated tumor remnants in all cases. Gross tumor volumes were higher on PET compared to MRI (*p = 0.032).

**Conclusion:**

Our data show that [^68^Ga]Ga-DOTA-TATE PET/CT is highly sensitive in revealing tumor remnants in patients with meningioma WHO grade 1. Negative PET findings were associated with a higher progression-free survival, thus improving surveillance. In patients with tumor remnants, additional PET can optimize adjuvant radiotherapy target planning of surgically resected meningiomas.

**Supplementary information:**

The online version contains supplementary material available at 10.1007/s00259-023-06400-3.

## Introduction

Meningiomas account for up to 40% of all intracranial tumors, representing the most frequently encountered primary brain tumors [1]. These lesions are classified into three grades based on histological and molecular criteria defined by the 2021 World Health Organization (WHO) classification of central nervous system tumors [2]. Microsurgical tumor resection with or without adjuvant radiation therapy (stereotactic radiosurgery or fractionated radiotherapy) is the established treatment of choice for the majority of symptomatic, space-occupying, or growing meningiomas [3]. Since the extent of resection (EOR) has been shown to correlate with the risk of recurrence, surgery aims to achieve an optimal balance between maximal safe tumor removal and preservation of neurological function [4, 5]. Recent outcome data from the NRG Oncology/RTOG 0539 trial prospectively validated favorable disease control rates and outcomes with a 10-year progression-free survival of up to 88% in patients receiving gross total tumor resection and observation [6]. However, the importance of postoperative radiotherapy, especially in patients undergoing incomplete tumor resection, remains a matter of debate [6]. Even in “benign” meningiomas WHO grade 1, a substantial subset of patients develops mid-to-long-term tumor recurrence or progressive disease and can even display atypical or malignant transformation at recurrence [2, 7, 8], prompting more aggressive therapy including re-resection, radiotherapy, and experimental pharmacological therapies in refractory cases [9, 10]. Early and accurate prediction of tumor recurrence in patients with meningioma WHO grade 1 therefore remains a pivotal component of clinical management, impacting recommendations for frequency of surveillance scans as well as indication and target volume delineation for adjuvant radiotherapy.

Historically, Simpson grading (SG) has been used as an intraoperative estimation for EOR; however, accuracy and prognostic significance of SG remain controversial [11–14]. Contrast-enhancing MRI is nowadays routinely used to assess EOR and serves as the established imaging modality for further surveillance scans during postoperative follow-up [3, 15, 16]. Nonetheless, structural imaging modalities such as MRI or CT scans have their limitations, especially in discriminating between viable tumor tissue, scars or post-therapeutic reactive changes, and struggle to estimate bony tumor involvement. In this context, functional imaging modalities such as the positron emission tomography (PET) are being increasingly applied to provide additional diagnostic information [17].

Somatostatin receptor type 2 (SSTR2) is ubiquitously expressed in up to 100% of meningiomas and can be addressed by radiolabeled SSTR2 ligands such as [^68^Ga]Gallium-DOTA-TATE, [^68^Ga]Gallium-DOTA-TOC, and most recently [^18^F]SiTATE [18, 19]. Biopsy-controlled studies have validated SSTR2 PET/CT as a highly sensitive tool for detecting meningioma tissue and distinguishing between tumor and tumor-free tissue, which is superior to MRI [20–23]. This enables differential diagnosis of MR morphologically ambiguous lesions [24], provides an excellent opportunity to assess the extent of resection after surgery [23, 25], and importantly also improves tumor delineation for radiation planning [26–29]. In this context, new grading systems for EOR incorporating postoperative PET/CT or PET/MRI findings have already been proposed, but prospective validation is still lacking, and prognostic relevance of postoperative SSTR2 PET/CT for tumor recurrence ultimately remains unclear [30].

In this single-center study, we describe a prospectively collected cohort of 46 patients with 49 meningiomas WHO grade 1 who were treated with microsurgical tumor resection and received postoperative [^68^Ga]Ga-DOTA-TATE PET/CT in addition to routinely used postoperative MRI to assess tumor residuals. We aim to prospectively evaluate the prognostic value of tumor remnants indicated by PET, compare it with postoperative MRI assessments, and discuss implications for adjuvant radiotherapy planning.

## Methods

### Study population

We conducted a single-center observational prospective study at the Department of Neurosurgery of the Ludwig-Maximilians-University in Munich, Germany. Study protocol and design were approved by the Institutional Review Board of the Ludwig-Maximilians-University in Munich, Germany (18-007), and patients’ informed consent was obtained. STROBE guidelines were followed whenever applicable (Supplementary Table 1). Patients were included based on the following criteria: (1) tissue-based diagnosis of CNS WHO grade 1 meningioma according to the 2016 WHO classification of CNS tumors [31] (2021 classification changes [32] did not impact the tumor classification in this cohort); (2) first-line treatment consisting of microsurgical tumor resection; (3) pre- and postoperative MRI < 6 months after surgery available for review; and (4) postoperative [^68^Ga]Ga-DOTA-TATE PET/CT scans < 6 months after surgery available for review. Patients were consecutively treated between 06/2016 and 09/2017 at our institution, and no further inclusion criteria were applied to avoid introduction of confounders. Patients were excluded from enrollment in case of severe renal insufficiency or gadolinium allergy, and excluded from analyses if follow-up time was less than 6 months. No further exclusion criteria were defined. Interim data on this prospective cohort was previously published by our group [25]. Demographics and clinical information, histopathologic diagnostics, treatment specifics, imaging, and outcome data were collected. Treatment decisions after surgery and in case of tumor progression/recurrence were based on multidisciplinary tumor board recommendations and patient preference. In detail, the evaluation of adjuvant treatment included all available data comprising baseline patient characteristics such as age and performance scores, pre- and postoperative MRI findings, tumor localization, histology and molecular markers, and intraoperative assessments. PET findings did not impact treatment decisions.

### Tumor resection and radiation therapy

Microsurgical tumor resection was performed by experienced surgeons with additional intraoperative ultrasound, neuromonitoring, and neuronavigation (BrainLab®, Munich, Germany). EOR was assessed by the operating surgeon according to Simpson’s definition of EOR in meningioma [33] and was documented immediately after surgery prior to acquisition of postoperative imaging.

For radiation therapy, patients received fractionated stereotactic radiotherapy (FSRT) as previously described [34]. For target volume delineations, both MRI including contrast-enhanced T1-weighted, non-contrast-enhanced T1-weighted, and T2-weighted sequences and [^68^Ga]Ga-DOTA-TATE PET/CT were used to define respective gross tumor volumes (GTVs) and identify the dural tail or any bone infiltration. The fusion of both GTVs was expanded 2 mm solely along the dura and the area of the skull base to obtain the clinical target volume (CTV). A uniform 3-mm expansion of the CTV was used to create the final planning target volume (PTV).

### Imaging

Postoperative imaging included contrast-enhanced T1-weighted MRI and [^68^Ga]Ga-DOTA-TATE PET/CT within 6 months after surgery. Further surveillance scans were obtained per current guidelines every 1–2 years or following clinical deterioration [3]. Imaging was reviewed by both experienced neurosurgeons and neuroradiologists. Definitions of tumor remnants, local tumor recurrence, or progression on MRI were based on contemporary guidelines of the Response Assessment in Neuro-Oncology Working Group [16]. In detail, standardized imaging protocols were applied including gadolinium-enhanced T1-weighted, non-contrast-enhanced T1-weighted, and T2-weighted imaging. Tumor remnants were defined as remaining bidimensional contrast-enhancing lesions in the region of the resection cavity. Tumor progression was defined as any progressive contrast enhancement on gadolinium-enhanced T1-weighted imaging compared to the baseline postoperative MRI. Tumor recurrence was defined as any new contrast enhancement in cases of complete resection according to baseline postoperative MRI.

[^68^Ga]Ga-DOTA-TATE PET/CT was performed using a Siemens biograph 64 PET/CT (Siemens Medical Solutions) as previously described [22, 25]. In short, ^68^Gallium-DOTA-Tyr3-Octreotate ([^68^Ga]Ga-DOTA-TATE), a radioligand targeting SSTR2, was applied for PET imaging and fused with contrast-enhanced CT scans. The manufacturer’s software (syngo.via; Siemens Healthcare) was used to analyze the reconstructed and fused PET/CT images. The PET mean and maximum standardized uptake values (SUV_mean_, SUV_max_) were assessed. An SUV_max_ > 2.3 in the region of interest served as a histologically verified cut-off for the detection of meningioma tissue and was therefore used to define positive PET findings [20]. Negative PET findings were correspondingly defined as an SUV_max_ < 2.3. In such positive areas, the biological tumor volume (BTV) was determined by semiautomatic threshold-based delineation. In case of tumor recurrence, postoperative PET was fused with the latest MRI image (Hermes Hybrid Viewer PDR 5.1.1) to allow correlation between the location of tumor recurrence and the location of tumor remnant according to postoperative PET. Significant correlation was defined as any overlap present between tumors on both imaging modalities in contrast to two distinct lesions without any overlap.

### Statistical analysis

As a primary endpoint, associations between positive postoperative PET findings with tumor recurrence during follow-up period were determined with a chi-squared test and Fisher’s exact test. Sensitivity, specificity, and predictive values were analyzed based on each respective tumor. MRI findings and Simpson gradings were analyzed accordingly. Categorical variables are expressed in absolute numbers and percentages. For survival analyses, Kaplan–Meier survival estimates were generated, and log-rank tests were calculated to compare tumor recurrences in patients. Patients were followed until data cut-off (December 1, 2022) or death. Individuals with a follow-up time of less than 6 months were excluded from survival analyses. Patients lost to follow-up were censored at day of last follow-up. The date of diagnosis was set as the date of microsurgical tumor resection. Progression-free survival was defined as the interval from diagnosis to radiographic progression. Statistical analyses on survival data were performed as post hoc analyses on the above outlined prospectively collected patient cohort. In this context, no sample size calculations or success thresholds were defined a priori.

Comparison of baseline variables between patients with positive or negative PET was performed using the chi-squared test and Fisher’s exact test for two or more categorical variables. The D’Agostino-Pearson omnibus normality test was utilized to test for normal distribution and equal variance in continuous data. The unpaired Student *t*-test was used to assess differences between two groups in case of parametric data, and the Mann–Whitney *U*-test was calculated for nonparametric variables. For paired data such as time intervals between different imaging modalities in each respective patient, the paired *t*-test and Wilcoxon matched-pairs signed rank test were utilized. Numerical data are described as mean ± standard error of the mean, and range is given, if not indicated otherwise. Statistical analyses were performed using a standard software package (SPSS Statistics version 25). The significance level was set at *p* ≤ 0.05.

## Results

### Patient and tumor characteristics

**Fig. 1:**
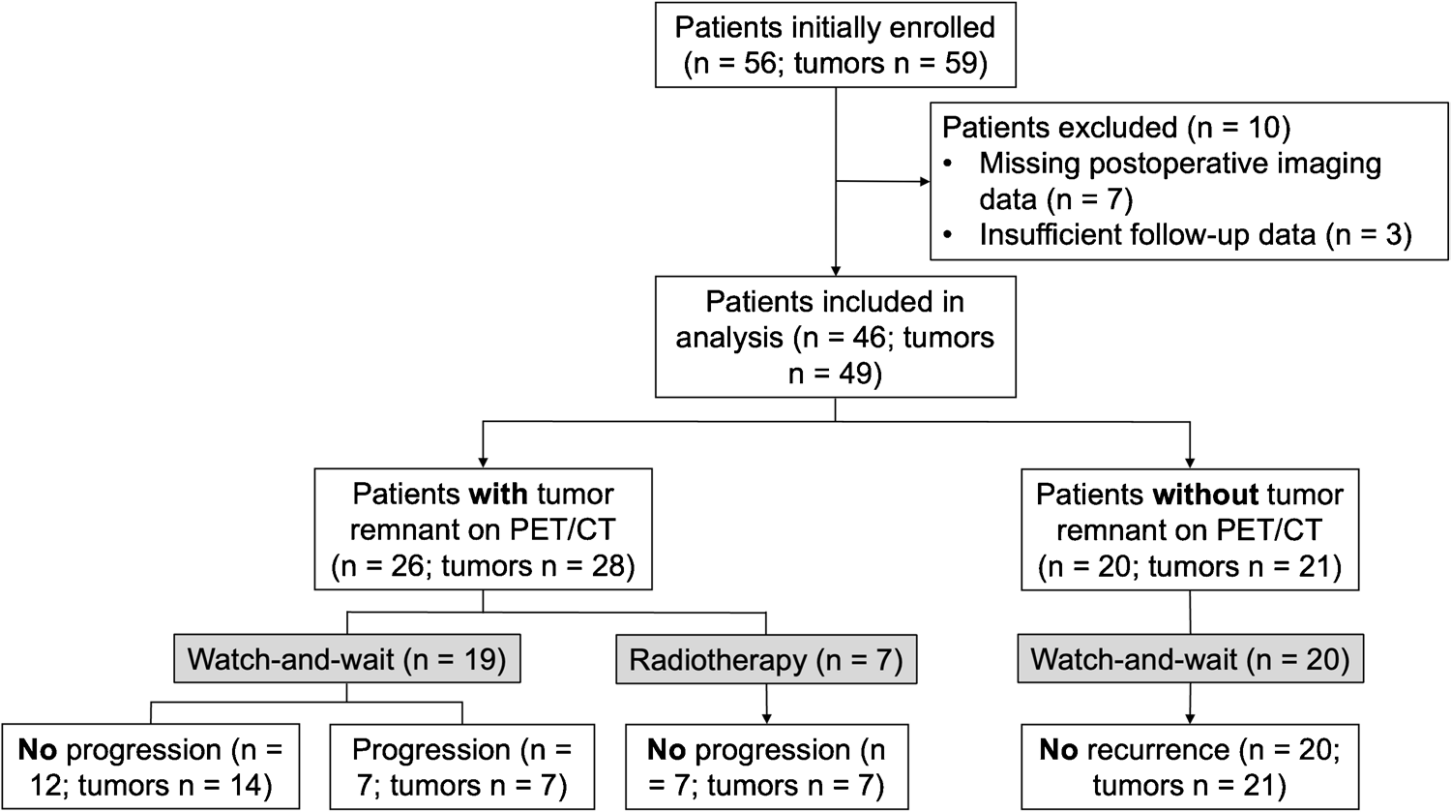
Consort diagram for patient selection. Schematic representation reporting enrollment, patient selection for analyses, and follow-up for the entire patient cohort with meningioma WHO grade 1 undergoing microsurgical tumor resection at the Centre for Neuro-Oncology at the Ludwig-Maximilians-University School of Medicine (*n* = 46)

A total of 56 patients were initially enrolled in the study with seven patients being ultimately excluded due to missing postoperative MRI. After further exclusion of three patients due to insufficient follow-up data, 46 patients presenting with 49 distinct tumors fulfilled the inclusion criteria, including one patient with two and one patient with three tumors (Fig. 1). In these patients, all 49 tumors were resected and diagnosed as meningioma WHO grade 1 according to the 2016 and 2021 WHO classification. Histologically, transitional meningiomas were most frequently encountered in 30 of 49 tumors (61%), followed by meningothelial tumors in nine of 49 tumors (18%) and fibroblastic tumors in five of 49 cases (10%). Microcystic (2 of 49, 4%), angiomatous (2 of 49, 4%), and secretory (1 of 49, 2%) meningiomas were rare. The mean age at diagnosis was 57.8 ± 1.7 years (range: 31–79 years) with a male-to-female ratio of 1:1.7. The majority of tumors were postoperatively followed via a watch-and-wait approach with surveillance scans every 1–2 years according to current standard of care (42 of 49 tumors, 86%) [3]. In the remaining seven of 49 tumors (14%) with SG IV and residual tumor on postoperative PET and MRI, postoperative fractionated stereotactic radiotherapy was provided (Table 1). The median follow-up in the entire patient cohort was 52 months (range: 10–73 months).

**Table 1:** Baseline characteristics for patients according to postoperative PET/CT findings

		PET/CT +	PET/CT −	Total	*p*-value
Overall patients, *n* (%)		26 (57%)	20 (43%)	46 (100%)	
Overall tumors, *n* (%)		28 (57%)	21 (43%)	49 (100%)	
Age, years	Mean	57.9 ± 2.4	57.7 ± 2.4	57.8 ± 1.7	0.950
Sex, *n* (%)	Female	13 (45%)	16 (55%)	29 (63%)	0.233
	Male	11 (65%)	6 (35%)	17 (37%)	
Tumor localization, *n* (%)	Skull base	10 (48%)	11 (52%)	21 (43%)	0.065
	Convexity	7 (47%)	8 (53%)	15 (30.5%)	
	Falcine/parasagittal	11 (85%)	2 (15%)	13 (26.5%)	
Histology, *n* (%)	Transitional	18 (60%)	12 (40%)	30 (61%)	0.298
	Meningothelial	4 (44%)	5 (56%)	9 (18%)	
	Fibroblastic	4 (80%)	1 (20%)	5 (10%)	
	Other	2 (40%)	3 (60%)	5 (10%)	
Simpson grade, *n* (%)	SG I	11 (52%)	10 (48%)	21 (43%)	*0.007
	SG II	4 (31%)	9 (69%)	13 (26.5%)	
	SG III	2 (50%)	2 (50%)	4 (8%)	
	SG IV	11 (100%)	0 (0%)	11 (22.5%)	
Residual tumor on postoperative MRI, *n* (%)	Yes	18 (86%)	3 (14%)	21 (43%)	*0.001
	No	0 (36%)	18 (64%)	8 (57%)	
Adjuvant treatment, *n* (%)	Watch-and-wait	21 (75%)	21 (100%)	42 (86%)	*0.015
	Radiotherapy	7 (25%)	0 (0%)	7 (14%)	

Characteristics are given for all patients with meningioma, CNS WHO grade 1 (*n* = 46 with *n* = 49 tumors), patients with tumor remnants according to postoperative [^68^Ga]Ga-DOTA-TATE PET/CT (*n* = 26), and those without any residual tumor (*n* = 20). Abbreviations: *PET/CT* + residual tumor on PET/CT imaging; *PET/CT-* no residual tumor on PET/CT imaging

### Imaging and extent of resection

Meningiomas were most commonly located at the skull base (21 of 49 tumors, 43%), followed by convexity meningiomas (15 of 49 tumors, 31%) and falcine/parasagittal lesions (13 of 49 tumors, 27%). The median time interval between tumor resection and image acquisition was not significantly different for MRI and PET scans (4 days, range: 1–140 days versus 27 days, range: 1–107 days; *p* = 0.256). Residual tumor could be demonstrated in 28 of 49 meningiomas (57%) according to postoperative PET, whereas 21 of 49 (43%) tumors revealed no remnants in PET. Twenty-one of 49 lesions (43%) showed tumor remnants on postoperative MRI. According to the surgeon’s estimate, SG I or II was achieved in 34 of 49 lesions (69%), whereas incomplete resection corresponding to SG III or IV was provided in the remaining 15 of 49 cases (31%).

### Outcome

**Fig. 2:**
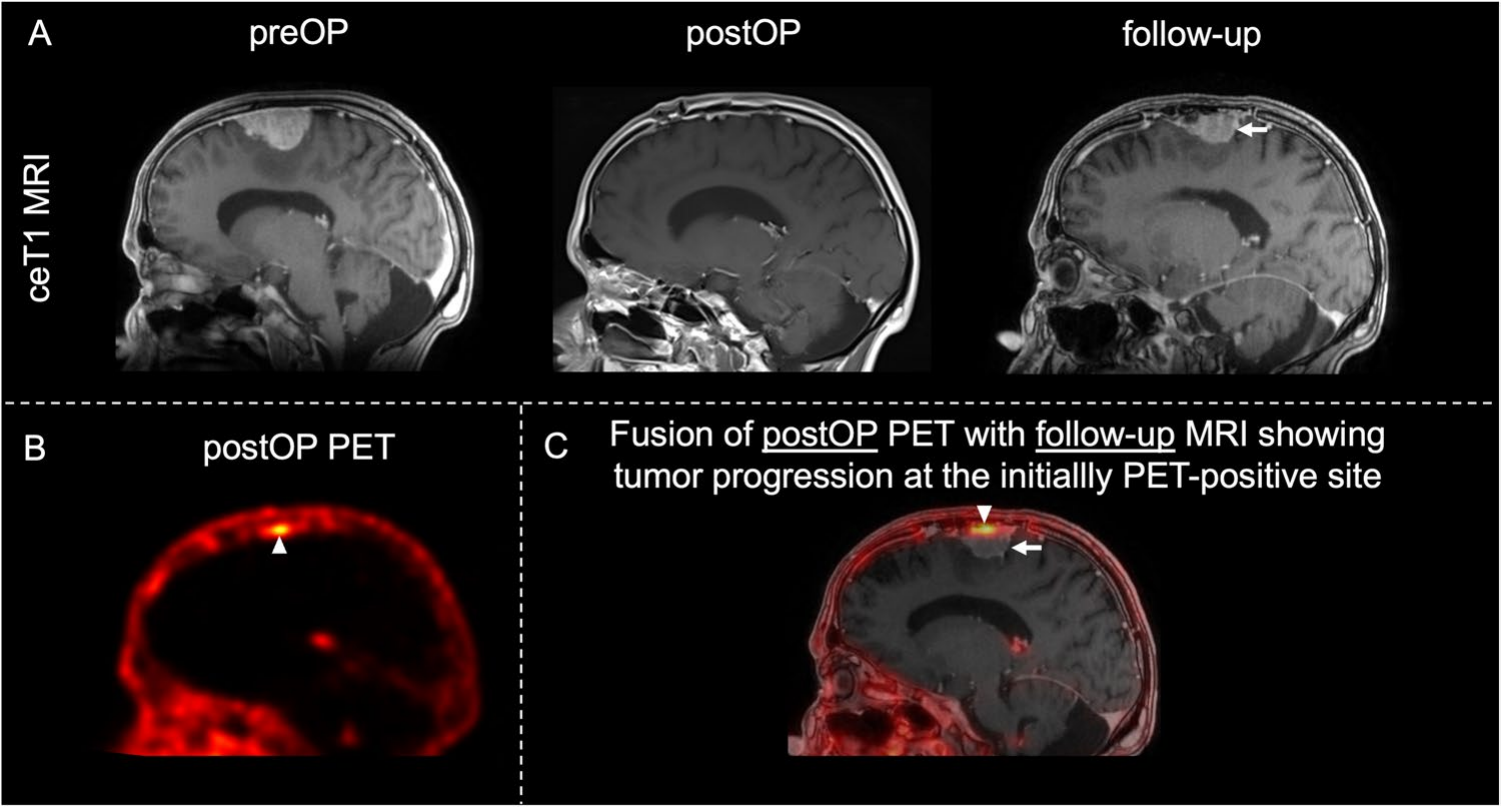
Case example of tumor progression. MRI and PET imaging in patients with tumor progression on PET. **A** Preoperative and postoperative sagittal contrast-enhancing T1-weighted MRI demonstrating homogenous contrast enhancement of a left hemispheric convexity meningioma with no apparent residual tumor after resection. Follow-up MRI showing consequent tumor recurrence at the same site (arrow). **B** Sagittal postoperative [^68^Ga]Ga-DOTA-TATE PET/CT scan of the same patient demonstrating residual tumor after resection (arrowhead). **C** Fusion images of postoperative PET scans and latest contrast-enhancing T1-weighted follow-up MRI showing a correlation between residual tumor on PET (arrowhead) and tumor progression on MRI (arrow). Abbreviations: *ceT1* contrast-enhancing T1-weighted MRI; *preOP* preoperative; *postOP* postoperative

The median overall survival (OS) was not reached with a median follow-up of 52 months (range: 10–73 months). The median recurrence/progression-free survival (PFS) was 66 months (range: 10–73 months), with a 3-year progression-free survival rate of 100%. Tumor progression was observed in seven of 46 patients (15%) and seven of 49 tumors (14%), and was located falcine/parasagittal in three of seven cases (43%), at the convexity in three of seven cases (43%), and at the skull base in one of seven cases (14%). Furthermore, localization of tumor progress on MRI showed a 100% correlation with regions of tracer uptake on postoperative PET being indicative for tumor remnants (Fig. 2A, B). Of note, postoperative PET demonstrated additional regions with values above the SUV_max_ threshold in these patients without apparent tumor progression on latest MRI (Fig. 2C).

### Predictors for outcome

#### PET

Excitingly, all patients with negative postoperative PET remained without recurrence (20 of 20 patients, 21 of 21 tumors, 100%). In patients with residual tumor on postoperative PET, seven of 28 tumors (25%) progressed, and 21 of 28 tumors (75%) remained progression-free. Of note, seven of these 21 PET-positive and progression-free tumors received postoperative radiotherapy (Fig. 1). Accordingly, tumor remnants on postoperative PET were significantly associated with tumor progression (*p = 0.015). The sensitivity of postoperative PET for tumor progression was 100% and specificity reached 50%. More importantly, the negative predictive value, meaning patients without tumor remnants on postoperative PET remaining recurrence-free during follow-up, was 100%. For patients with tumor remnants on postoperative PET, the positive predictive value to develop tumor progression was 25%. To account for different treatment strategies including adjuvant radiotherapy, we only analyzed tumors homogenously treated with a watch-and-wait approach (42 of 49, 86%). Here, the negative predictive value was 100%, and the positive predictive value increased to 33% with a sensitivity and specificity of 100% and 60%, respectively.

**Fig. 3:**
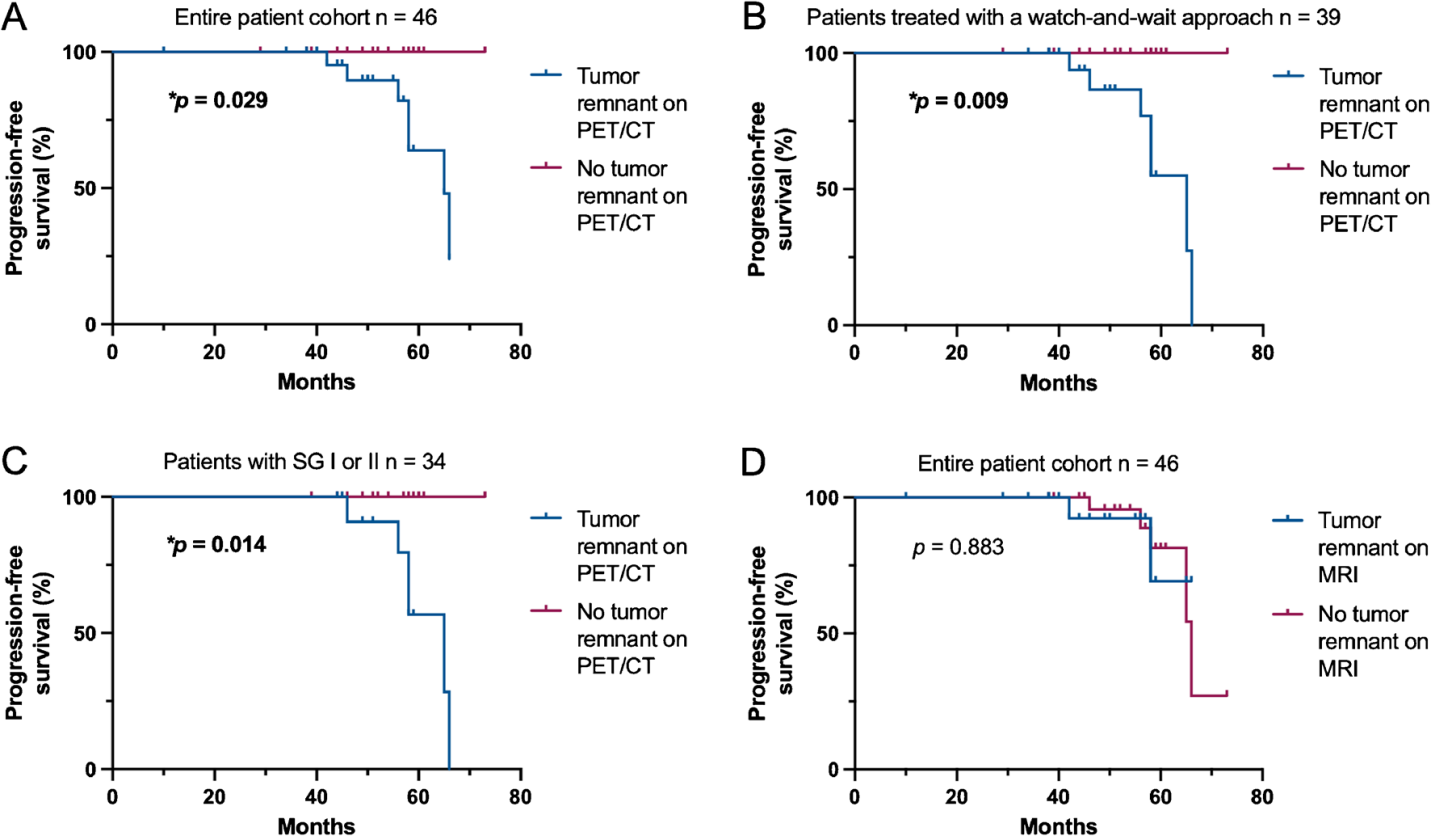
[^68^Ga]Ga-DOTA-TATE PET/CT is prognostic for tumor progression in meningioma WHO grade 1. **A**–**D** Kaplan–Meier estimates of progression-free survival in patients with meningioma WHO grade 1 treated with microsurgical tumor resection in the entire patients cohort (*n* = 46, **A** and **D**), patients receiving postoperative watch-and-wait only (*n* = 39, **B**), and patients in which SG 1 and 2 were achieved (*n* = 34, **C**). Curves are displayed for patients with (blue) and without (red) tumor remnants on postoperative [^68^Ga]Ga-DOTA-TATE PET/CT (**A**–**C**) and on postoperative MRI (**D**). Tick marks indicate censored patients. Abbreviations: *SG* Simpson grades

When comparing patients with and without residual tumor on postoperative PET, age, sex distribution, tumor localization, and histologies were comparable in both groups (Table 1). As suspected, SG was significantly higher in patients with residual tumor on PET, and MRI findings of residual tumor were more frequently encountered (*p* = 0.007 and *p* = 0.001, respectively). Postoperative radiotherapy was more frequently administered in patients with residual tumor on PET in accordance with current guidelines (*p* = 0.015). Importantly, PFS was significantly higher in patients with negative postoperative PET scans in comparison to those with detectable tumor remnants (no progression versus 65 months, *p = 0.029; Fig. 3A). The same held true when only comparing patients observed with a watch-and-wait approach (*p = 0.009; Fig. 3B).

Next, we wanted to evaluate the importance of PET findings in case of “complete” tumor resections as judged by the operating surgeon and corresponding to SG I and II (*n* = 34). Here, tumor residuals on PET proved to be strongly associated with tumor progression (*p = 0.004), and a positive predictive value of 40% was reached, while the negative predictive value remained 100%. Again, PFS was significantly higher in patients with negative PET findings (*p = 0.014; Fig. 3C).

In a next step, we analyzed whether PET parameters in patients with residual tumor on postoperative PET differed between patients showing tumor recurrence and those who presented with stable disease. Here, SUV_max_, SUV_mean_, and biological target volume were similar between both groups (*p* = 0.770, *p* = 0. 0.874, *p* = 0.770, respectively; Supplementary Table 2). There was no difference between the follow-up period for both groups (*p* = 0.13).

#### MRI

Five of seven patients with tumor recurrence showed no evidence of tumor remnants on postoperative MRI. Fisher’s exact test showed no association between tumor remnants on MRI and tumor progression/recurrence (*p* = 0.683). Sensitivity and specificity were 29% and 55%, respectively, with a positive predictive value of 10% and a negative predictive value of 82%. Exclusion of patients who received postoperative radiotherapy resulted in a positive predictive value of 14%. Detection of tumor remnants on MRI was not associated with less favorable outcome (*p* = 0.883; Fig. 3D).

#### Simpson grading

Simpson grading was not associated with tumor progression/recurrence (*p* = 0.414). Six of 34 meningiomas (18%) displayed tumor recurrence after SG I or II resections, and one of 15 patients (7%) had tumor progression after SG IV resection corresponding to a sensitivity and specificity of 14% and 67%, respectively. Of note, this patient did not receive postoperative radiotherapy. These findings held true when comparing only patients homogenously treated with a watch-and-wait approach (*n* = 42, *p* = 0.999). “Complete” tumor resection (SG I and II) was not associated with improved PFS (*p* = 0.590).

### Implications for radiotherapy

**Fig. 4:**
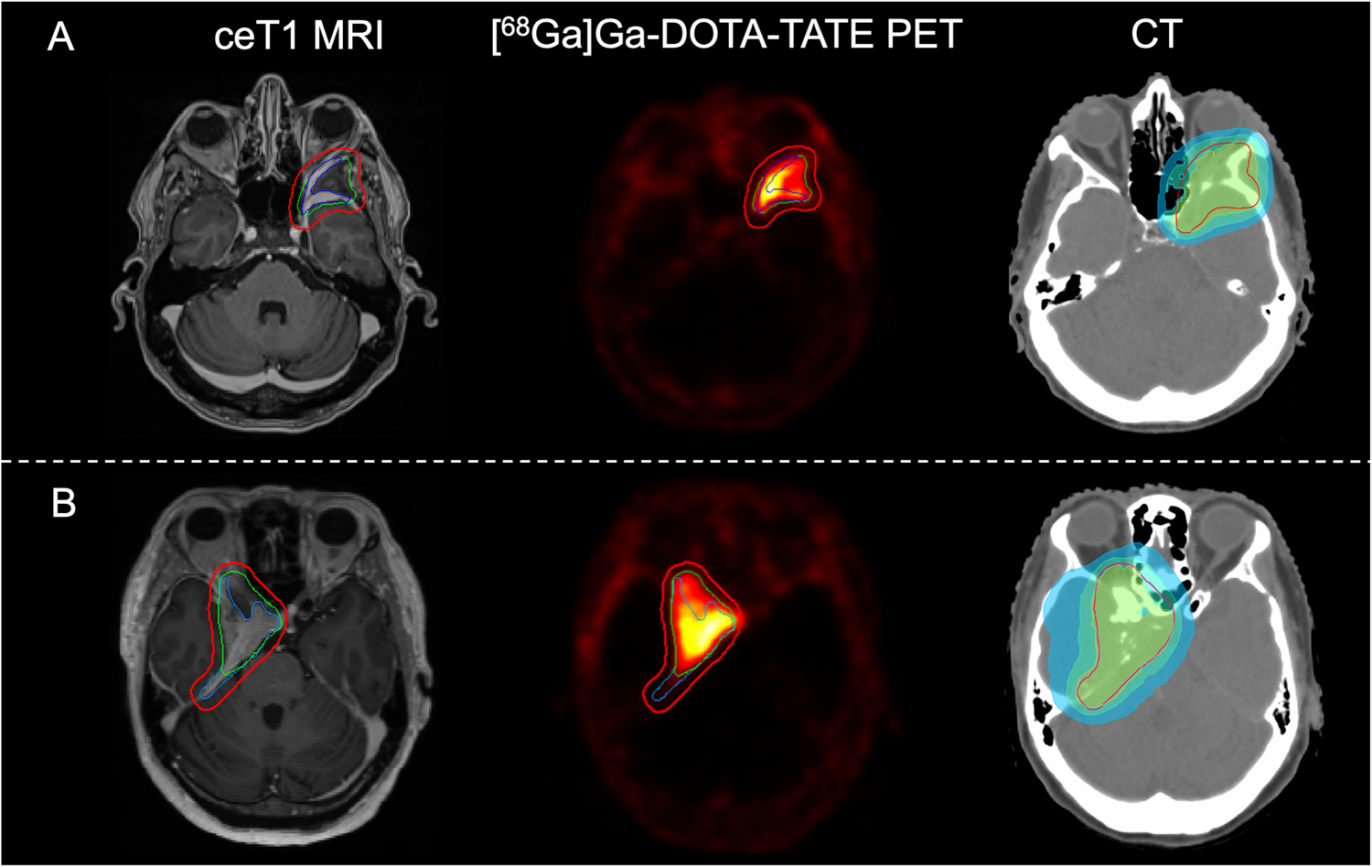
Radiotherapy planning in patients with incomplete tumor resection. **A**, **B** Postoperative contrast-enhancing T1-weighted MRI, [^68^Ga]Ga-DOTA-TATE PET scans, and planning CT depicted for two patients with tumor remnants on MRI and PET. GTV on MRI is delineated in blue; GTV on PET is delineated in green. Fusion of both with additional expansion resulting in the respective PTV is depicted in red. Radiation plan based on PTV is shown on simulation CT, performed immediately before the start of radiotherapy. **A** Patient with a left-sided sphenoid wing meningioma showing tumor remnants on along the dura of the sphenoid wing and lateral orbital wall on MRI but also demonstrating bony tumor involvement of the sphenoid wing on PET. **B** Patient with a right-sided skull base meningioma and tumor remnants along the middle cranial fossa, sellar region, and optic canal on MRI showing additional tumor tissue on PET. Abbreviations: *ceT1* contrast-enhancing T1-weighted

Seven of 46 patients (14%) were treated with FSRT. In six of seven patients, radiotherapy planning data were available. Here, GTV as delineated on PET was significantly higher in comparison to MRI GTV (12.1 ± 3.3 cm^3^ versus 7.8 ± 2.7 cm^3^; *p = 0.032; Table 2). PET was especially helpful in determining bony tumor involvement, leading to a different PTV in five of six patients (83%; Fig. 4A, B).

**Table 2:** Comparison of gross tumor volumes based on MRI and PET/CT for radiotherapy planning

	MRI GTV (cm^3^)	PET/CT GTV (cm^3^)	Volume difference (cm^3^)	*p*-value
Patient 1	12.8	17.1	4.3	Not applicable
Patient 2	16.9	24.4	7.5	
Patient 3	5.2	4.7	∣-0.5∣	
Patient 4	6.0	15.0	9.0	
Patient 5	1.7	3.4	1.8	
Patient 6	4.2	7.7	3.5	
Total	7.8 ± 2.7	12.1 ± 3.3	4.4 ± 1.3	*0.032

Gross tumor volumes as defined by MRI and PET/CT as well as absolute volume differences are given for each respective patient treated with fractionated stereotactic radiotherapy after undergoing tumor resection (available in 6/7 patients). Paired *t*-test was used for comparison of volumes. Abbreviations: *GTV* gross tumor volume

## Discussion

Somatostatin receptor PET imaging has been shown to be highly sensitive in detecting tumor remnant after microsurgical tumor resection in patients with intracranial meningioma [23, 25]. However, the prognostic importance of residual tumor as indicated by [^68^Ga]Ga-DOTA-TATE PET/CT is so far unclear. Based on a prospective cohort of 46 patients with 49 meningiomas WHO grade 1 undergoing microsurgical tumor resection, we aimed to elaborate on the clinical relevance of tumor remnants on [^68^Ga]Ga-DOTA-TATE PET/CT.

We could show that [^68^Ga]Ga-DOTA-TATE PET/CT suggestive for residual tumor after resection was prognostic for tumor progression and associated with lower progression-free survival. In addition, our data demonstrated a strong correlation between the localization of tumor progression and postoperative tumor remnants as indicated by PET. Importantly, all patients with negative postoperative PET findings after resection remained recurrence-free until data base closure. On a cautionary note, patients with and without tumor remnants on postoperative PET were heterogeneously treated as 7 patients in our study population received adjuvant FSRT after PET findings were suggestive for tumor remnants and followingly remained progression-free. However, in our subgroup analysis of patients homogeneously treated with a watch-and-wait approach after tumor resection, PET remained prognostic for tumor progression with an even higher positive predictive value of 33%. In comparison, both SG and MRI proved insufficient for distinguishing patients who developed tumor recurrence or progression from those who did not.

Age, sex distribution, and histology in our study population were characteristic for meningiomas, and different tumor localizations were evenly distributed. A tumor recurrence rate of 14% at 4.3 years was in line with previous publications reporting recurrence rates of 6–23% 5 years after gross total tumor resection [6, 35]. As the study population exclusively consisted of “benign” WHO grade 1 meningiomas, one would expect an increased tumor recurrence or progression rate with longer follow-up [8]. In this context, one would assume that the positive predictive value of PET findings indicating residual tumor in predicting tumor progression may increase with a longer follow-up period. Of note, unspecific tracer uptake has been previously described during the first weeks after glioma resection [36]. In [^68^Ga]Ga-DOTA-TATE PET/CT after meningioma resection, possible transient tracer uptake has not been demonstrated yet. However, image acquisition should be performed not within the first 2 weeks after surgery to avoid possible unspecific tracer uptake and false-positive findings.

Beside histopathologic and molecular tumor characteristics, early and accurate assessment of residual meningioma tissue remains of clinical importance, as it potentially changes adjuvant patient management. In this regard, information about tumor remnants on PET not only helps clinicians in accurately prognosticating the risk of progression/recurrence for their patients but could potentially impact the decision to start adjuvant radiotherapy. Based on our results, every third patient with PET/CT findings indicative for tumor remnant and treated with surgery and observation developed local tumor progression during follow-up. Hence, we suggest that postoperative SSR PET should at least be added to the portfolio of postoperative imaging modalities to optimize assessment of EOR and subsequently guide clinical decision-making in patients with otherwise borderline radiotherapy indications. Patients with negative PET are highly unlikely to develop tumor recurrence and can therefore be monitored with extended surveillance scan intervals. Patients with positive PET findings should be monitored closely with reduced surveillance scan intervals for timely detection of tumor progress. Importantly, however, adjuvant radiotherapy indications should be critically evaluated and not solely based on postoperative PET findings but rather on a case-by-case basis, given the overall low tumor progression rate in patients with meningioma WHO grade 1. Especially for tumor localizations like parafalcine lesions with involvement of the superior sagittal sinus or skull base meningiomas, where complete tumor resection is often difficult to achieve, postoperative PET seems to be a helpful add-on to better estimate EOR and the risk for tumor progression. Adjuvant radiotherapy can be considered in these patients. This is particularly important, as recurrent meningiomas have an increased risk of progression and the potential to display atypical or malignant transformation at recurrence, necessitating more aggressive therapy associated with increased morbidity [2, 7]. Furthermore, early re-resection of tumor residuals should be carefully evaluated; however, complex localizations and preservation of neurological function seem to limit its benefit [37]. On a side note, one has to assume that in light of improving imaging modalities for assessment of EOR, many so-called local tumor recurrences, meaning recurrences without prior evidence of tumor remnants on MRI, rather depict slow tumor progressions when additional PET scans are taken into account, and careful consideration is warranted when reporting on such outcomes.

Critically, improved target volume delineation based upon PET scans may come with an increased disease control rate, but also lower toxicities due to personalized dose distribution [27, 38]. In our cohort, patients received radiotherapy based on both MRI and PET-guided therapy planning, and all of them remained progression-free. GTVs delineated based on PET were significantly higher than MRI GTV and superior in detecting bony tumor involvement, providing a rationale to include PET for radiation planning. Additionally, PET enables detection of distant foci otherwise not evident on MRI scans which can consequently be included in the respective target volume planning. Simultaneous somatostatin receptor PET/MRI, combining high soft tissue resolution structural imaging with metabolic and cellular features from PET/CT, may be an even more accurate option for assessing postoperative tumor remnants [39]. However, its lack of availability hinders the widespread use of such devices. Moreover, given the fact that the skull serves as excellent reference for the fusion process, PET/CT seems comparable to PET/MRI in cranial imaging.

Considering its daily application, ^68^Gallium-labeled somatostatin receptor ligands are associated with several limitations. Especially the ^68^Ge/^68^Ga generators, although enabling in-house production without need of an on-site cyclotron, currently remain cost intensive. The ligands show low activity amounts and a short half-life. Here, the [^18^F]SiTATE tracer provides a promising alternative that may advance the widespread use of SSTR ligands and overcome the drawbacks of ^68^Gallium-labeled ligands [17, 19, 40]. It is highly selective for the SSTR receptor subtype 2 with only minor or no affinity to SSTR types 1 and 3–5 [41]. Furthermore, the significantly lower positron energy of ^18^F compared to ^68^Ga (mean positron energy 0.25 vs. 0.83 MeV) leads to a better spatial resolution in PET imaging due to a shorter positron range (mean range 0.6 vs. 3.5 mm). The cyclotron-based synthesis of ^18^F allows for significantly higher activities per synthesis. In combination with the longer half-life compared to ^68^Ga (110 vs. 68 min), a higher number of patients can be examined per day, and transportation of [^18^F]SiTATE to other additional imaging sites is manageable (satellite principle) [42].

This, in turn, is relevant from a health economics’ perspective. As SSTR2 PET and its associated ligands become more cost-effective and can be offered on a widespread basis, a reduction in the frequency of postoperative surveillance scans via MRI considering its limitations in predicting tumor recurrence has to be evaluated. In this context, further cost-effectiveness analyses are warranted.

The limitations of our study included the limited sample size and different adjuvant treatment strategies that were applied in our patient cohort depending on extent of resection. No additional sample size calculations and success thresholds for survival data were defined a priori, possibly introducing bias. Large prospective studies are needed to validate our promising findings in this preliminary study and take postoperative treatment strategies into account.

Histological grading, DNA methylation profiling, and copy number analyses have been shown to predict the recurrence risk in meningioma, in addition to WHO grading and extent of resection, and were shown to be helpful in accurately identifying patients at high risk for tumor recurrence [43, 44]. Interestingly, studies could show that only 20% of all meningiomas WHO grade 1 demonstrated a methylation profile associated with higher risk for recurrence [45]. Most meningiomas WHO grade 1, therefore, remained at low risk for recurrence even when incorporating DNA methylation profiling. Nevertheless, missing molecular characterizations including DNA methylation profiling in our study cohort of meningiomas WHO grade 1 could possibly represent confounding factors.

Future studies will need to assess the prognostic relevance of [^68^Ga]Ga-DOTA-TATE PET/CT in higher grade meningiomas WHO grades 2 and 3. A previously published small retrospective analysis on patients with WHO grades 2 and 3 meningiomas undergoing radiotherapy only found metabolic tumor volume on PET prior to radiotherapy to be predictive for progression-free survival [46]. Especially in meningiomas WHO grade 2, where the role of adjuvant radiotherapy is still a subject of discussion when complete tumor resection is provided, accurate assessment of EOR and knowledge of its prognostic value will prove crucial for risk stratification and consequently optimal patient management [3]. In this regard, the question of watch-and-wait versus radiotherapy is currently being addressed by the ROAM/EORTC-1308 trial (ISRCTN71502099).

In conclusion, our data show that [^68^Ga]Ga-DOTA-TATE PET/CT is highly effective in revealing postoperative tumor residuals in patients with meningioma WHO grade 1, and superior to commonly used MRI and Simpson grading. Importantly, negative PET findings were strongly associated with a decreased risk for tumor progression and higher progression-free survival. In patients with tumor remnants, PET improved adjuvant radiotherapy planning for surgically resected meningiomas.

### Supplementary Information

Below is the link to the electronic supplementary material.

Supplementary file1 (DOCX 38 KB)

Supplementary file2 (DOCX 49 KB)

## Data Availability

The data presented in this study are available on request from the corresponding authors. The data are not publicly available due to the guidelines of the Institutional Review Board of the Ludwig-Maximilians-University in Munich.
