# Supplementary material for: Postoperative [68Ga]Ga-DOTA-TATE PET/CT imaging is prognostic for progression-free survival in meningioma WHO grade 1
Source: Eur J Nucl Med Mol Imaging. 2023 Aug 29;51(1):206–17. doi: 10.1007/s00259-023-06400-3 (PMC10684417; doi:10.1007/s00259-023-06400-3)
Supplement: Supplementary file 1 — Supplementary file1 (DOCX 38 KB) [file 259_2023_6400_MOESM1_ESM.docx]

**Supplementary Table 1: STROBE checklist for observational studies**

|  | Item No. | Recommendation | Page  No. | Relevant text from manuscript |
| --- | --- | --- | --- | --- |
| **Title and abstract** | 1 | (*a*) Indicate the study’s design with a commonly used term in the title or the abstract | 1,2 | See Title and abstract “prospective single-center study” |
|  |  | (*b*) Provide in the abstract an informative and balanced summary of what was done and what was found | 2 | See abstract |
| Introduction | | | |  |
| Background/rationale | 2 | Explain the scientific background and rationale for the investigation being reported | 3,4 | See introduction |
| Objectives | 3 | State specific objectives, including any prespecified hypotheses | 4 | “We aim to prospectively evaluate the prognostic value of tumor remnants indicated by PET-CT, compare it with postoperative MRI assessments and discuss implications for adjuvant radiotherapy planning.” |
| Methods | | | |  |
| Study design | 4 | Present key elements of study design early in the paper | 4,5 | “We conducted a single-center observational prospective study…” |
| Setting | 5 | Describe the setting, locations, and relevant dates, including periods of recruitment, exposure, follow-up, and data collection | 4,5 | See paragraph “study population” |
| Participants | 6 | (*a*) *Cohort study*—Give the eligibility criteria, and the sources and methods of selection of participants. Describe methods of follow-up  *Case-control study*—Give the eligibility criteria, and the sources and methods of case ascertainment and control selection. Give the rationale for the choice of cases and controls  *Cross-sectional study*—Give the eligibility criteria, and the sources and methods of selection of participants | 5 | See paragraph “study population” and figure 1  “Patients were included based on the following criteria: 1) tissue-based diagnosis of CNS WHO grade 1 meningioma according to the 2016 WHO classification of CNS tumors31 (2021 classification changes32 did not impact the tumor classification in this cohort); 2) first-line treatment consisting of microsurgical tumor resection; 3) pre- and postoperative MRI <6 months after surgery available for review; and 4) postoperative 68Ga-DOTATATE/PET-CT scans <6 months after surgery available for review.” |
|  |  | (*b*) *Cohort study*—For matched studies, give matching criteria and number of exposed and unexposed  *Case-control study*—For matched studies, give matching criteria and the number of controls per case |  | See above |
| Variables | 7 | Clearly define all outcomes, exposures, predictors, potential confounders, and effect modifiers. Give diagnostic criteria, if applicable | 5-7 | e.g., “Imaging was reviewed by both, experienced neurosurgeons and neuroradiologists. Definitions of tumor remnants, local tumor recurrence or progression on MRI were based contemporary guidelines of the Response Assessment in Neuro-Oncology Working Group.” and “An SUV > 2.3 served as a histologically verified cut-off for the detection of meningioma tissue.” |
| Data sources/ measurement | 8* | For each variable of interest, give sources of data and details of methods of assessment (measurement). Describe comparability of assessment methods if there is more than one group | 5-7 | See above |
| Bias | 9 | Describe any efforts to address potential sources of bias | 5, 11, 13 | “Patients were consecutively treated between 06/2016 and 09/2017 at our institution and no further criteria were applied to avoid introduction of confounders.”  “On a cautionary note, patients with and without tumor remnants on postoperative PET were heterogeneously treated as 7 patients in our study population received adjuvant FSRT after PET findings were suggestive for tumor remnants and followingly remained progression-free.” |
| Study size | 10 | Explain how the study size was arrived at | 6 | See above |

| Quantitative variables | 11 | Explain how quantitative variables were handled in the analyses. If applicable, describe which groupings were chosen and why | 6,7 | See paragraph “statistical analyses” |
| --- | --- | --- | --- | --- |
| Statistical methods | 12 | (*a*) Describe all statistical methods, including those used to control for confounding | 6,7 | See above |
|  |  | (*b*) Describe any methods used to examine subgroups and interactions | 6,7 | See above |
|  |  | (*c*) Explain how missing data were addressed | 6,7 | See above |
|  |  | (*d*) *Cohort study*—If applicable, explain how loss to follow-up was addressed  *Case-control study*—If applicable, explain how matching of cases and controls was addressed  *Cross-sectional study*—If applicable, describe analytical methods taking account of sampling strategy | 6,7 | See above |
|  |  | (*e*) Describe any sensitivity analyses | 6,7 | See above |
| Results | | | | |
| Participants | 13* | (a) Report numbers of individuals at each stage of study—eg numbers potentially eligible, examined for eligibility, confirmed eligible, included in the study, completing follow-up, and analysed | 7,8 | Figure 1 and results paragraph 1 |
|  |  | (b) Give reasons for non-participation at each stage | 7,8 | Figure 1 |
|  |  | (c) Consider use of a flow diagram | 7,8 | **Figure 1** |
| Descriptive data | 14* | (a) Give characteristics of study participants (eg demographic, clinical, social) and information on exposures and potential confounders | 7,8 | Table 1 + e.g., “Mean age at diagnosis was 57.8 ± 1.7 years (range: 31 - 79 years) with a male-to-female ratio of 1:1.7.” |
|  |  | (b) Indicate number of participants with missing data for each variable of interest | 8-11 | Indicated thoroughly in each respective results paragraph |
|  |  | (c) *Cohort study*—Summarise follow-up time (eg, average and total amount) | 8 | See paragraph *outcome* |
| Outcome data | 15* | *Cohort study*—Report numbers of outcome events or summary measures over time | 8-10 | Indicated thoroughly in each respective results paragraph |
|  |  | *Case-control study—*Report numbers in each exposure category, or summary measures of exposure | n.a. | n.a. |
|  |  | *Cross-sectional study—*Report numbers of outcome events or summary measures | n.a. | n.a. |
| Main results | 16 | (*a*) Give unadjusted estimates and, if applicable, confounder-adjusted estimates and their precision (eg, 95% confidence interval). Make clear which confounders were adjusted for and why they were included | n.a. | n.a. |
|  |  | (*b*) Report category boundaries when continuous variables were categorized | 7 | See statistical analyses |
|  |  | (*c*) If relevant, consider translating estimates of relative risk into absolute risk for a meaningful time period | n.a. | n.a. |

| Other analyses | 17 | Report other analyses done—eg analyses of subgroups and interactions, and sensitivity analyses | 9,10 | e.g.,” Next, we wanted to evaluate the importance of PET-CT findings in case of ‘complete’ tumor resections…” |
| --- | --- | --- | --- | --- |
| Discussion | | | | |
| Key results | 18 | Summarise key results with reference to study objectives | 11 | e.g.,” We could show that 68Ga-DOTATATE/PET-CT imaging suggestive for residual tumor after resection was prognostic for tumor progression…” |
| Limitations | 19 | Discuss limitations of the study, taking into account sources of potential bias or imprecision. Discuss both direction and magnitude of any potential bias | 13 | e.g.,” On a cautionary note, our sample size was limited, and different adjuvant treatment strategies were applied in our patient cohort depending on extent of resection…” |
| Interpretation | 20 | Give a cautious overall interpretation of results considering objectives, limitations, multiplicity of analyses, results from similar studies, and other relevant evidence | 12 | See discussion |
| Generalisability | 21 | Discuss the generalisability (external validity) of the study results | 12 | See discussion |
| Other information | |  | | |
| Funding | 22 | Give the source of funding and the role of the funders for the present study and, if applicable, for the original study on which the present article is based | 1 | “No targeted funding to report.” |
