# Supplementary material for: Postoperative [68Ga]Ga-DOTA-TATE PET/CT imaging is prognostic for progression-free survival in meningioma WHO grade 1
Source: Eur J Nucl Med Mol Imaging. 2023 Aug 29;51(1):206–17. doi: 10.1007/s00259-023-06400-3 (PMC10684417; doi:10.1007/s00259-023-06400-3)
Supplement: Supplementary file 2 — Supplementary file2 (DOCX 49 KB) [file 259_2023_6400_MOESM2_ESM.docx]

**Supplementary Table 2: [^68^Ga]Ga-DOTA-TATE PET/CT parameters depending on tumor recurrence**

Comparison of PET/CT parameters in case of residual tumor on postoperative PET/CT imaging in patients with and without tumor recurrence during follow-up. *Abbreviations*: *SUV_max_ –* maximum standardized uptake value; SUV_mean_ – mean standardized uptake value; *BTV –* biological target volume.

|  | **Tumor recurrence** | **No tumor recurrence** | ***p*-value** |
| --- | --- | --- | --- |
| **SUV_max_** | 4.91 (3.09-18.07) | 5.11 (2.52-31.86) | 0.770 |
| **SUV_mean_** | 3.67 (2.58-6.78) | 3.09 (2.43-9.33) | 0.874 |
| **BTV** | 2.3 (0.24-17.42) | 1.29 (0.1-27.3) | 0.770 |
